# “Drinkers Like Me”: A Thematic Analysis of Comments Responding to an Online Article About Moderating Alcohol Consumption

**DOI:** 10.3389/fpsyg.2022.780677

**Published:** 2022-03-14

**Authors:** Patricia Irizar, Jo-Anne Puddephatt, Jasmine G. Warren, Matt Field, Andrew Jones, Abigail K. Rose, Suzanne H. Gage, Laura Goodwin

**Affiliations:** ^1^Department of Psychology, Institute of Population Health, University of Liverpool, Liverpool, United Kingdom; ^2^Liverpool Centre for Alcohol Research, Liverpool Health Partners, Liverpool, United Kingdom; ^3^Department of Psychology, University of Sheffield, Sheffield, United Kingdom; ^4^School of Psychology, Liverpool John Moores University, Liverpool, United Kingdom

**Keywords:** social norms, cultural practices, thematic analysis, alcohol abstinence, alcohol consumption, qualitative

## Abstract

**Background:**

There has been media coverage surrounding the dangers of heavy drinking and benefits of moderation, with TV and radio presenter, Adrian Chiles, documenting his experience of moderating alcohol consumption in an online article for the Guardian. By analysing the comments in response to Chiles’ article, this study aimed to explore (i) posters’ (someone who has posted a comment in response to the article) attitudes or beliefs toward moderating alcohol and (ii) posters’ experiences of moderating or abstaining from alcohol.

**Method:**

A secondary qualitative analysis of online comments in response to an article about moderating alcohol consumption. Main outcome measures: Comments (*n* = 784) in response to a United Kingdom online news article about moderating alcohol consumption were extracted and inductive thematic analysis was used.

**Results:**

For aim one, two themes were developed; “general attitudes toward drinking” and “general attitudes toward reducing consumption”. These themes reflect negative perceptions of alcohol and issues around changing attitudes. For aim two, three themes were developed: “moderation vs. abstention”, “reflection on past drinking behaviours”, and “current drinking behaviours”. These themes represent posters’ experiences and implications changing their drinking habits.

**Conclusion:**

Our analysis provides a novel insight into perceptions and experiences of moderating or abstaining from alcohol. Alcohol is embedded within United Kingdom culture, creating difficulties for those who choose to moderate or abstain from alcohol. Our analysis highlights the need for public health to focus on shifting the current drinking culture, through clearer drinking guidelines and a wider availability of alcohol-free alternatives.

## Introduction

According to a recent United Kingdom (UK) survey of adult drinking behaviour, 28% of males and 14% of females reported drinking at hazardous or harmful levels (NHS Digital, 2018). Hazardous drinking refers to a quantity or pattern of consumption that places you at risk of adverse events (above 14 units per week – one United Kingdom unit equals 10 ml or 8 g of pure alcohol), and harmful drinking refers to consumption that results in adverse events (above 35/50 units per week for females/males) (Department of Health and Social Care, 2016). Although rates of hazardous and harmful drinking have decreased among young people since 2011 (and abstinence rates have increased), they continue to rise in older adults (Orchard, 2015; Bardsley et al., 2018; Oldham et al., 2019). Moreover, alcohol-attributable harm appears to be rising at a population-level, as seen through increased alcohol-related hospital admissions (Orchard, 2015; Holmes et al., 2019). This may reflect a national drinking culture in the United Kingdom whereby alcohol is integral to many practices, particularly social practices, as outlined by theories of practices (Ally et al., 2016; Meier et al., 2018). Interventions aimed at reducing alcohol-related harms must tackle the underlying culture. Therefore, understanding the attitudes toward, and experiences of, moderating consumption or abstinence plays a critical role in changing this heavy drinking culture.

There is limited research into the experiences of those who abstain or moderate alcohol consumption; understanding their motivations and successful strategies can aid the development of population-level interventions. Qualitative research of Australian adults attempting to moderate found that many experienced stigma for violating social norms, yet adapted their social practices by replacing alcohol with other drinks or choosing other social activities (Bartram et al., 2017a,b). Similar research has shown that abstainers are perceived by drinkers as a “threat to fun”, contributing to stigma (Cheers et al., 2020). A quantitative tool has been developed to measure attitudes toward abstainers, finding that heavier drinkers hold more negative attitudes toward non-drinkers, relating to the potentially detrimental social consequences (Regan and Morrison, 2013, 2017). Contrarily, interviews with moderators and abstainers identified a more positive experience, as they reported “feeling good in the body” and “feeling safe and secure” (Graber et al., 2016). A United Kingdom survey of high-risk drinkers, trying to moderate, stated their main reasons as weight loss, health and cost (Beard et al., 2017). There are compelling health and economic reasons to abstain or moderate alcohol consumption, yet the social consequences may deter people.

Online discussion forums have yielded a rich source of naturalistic secondary qualitative data for health research (Kozinets, 2015). There are advantages to this unique methodology in comparison to standard data collection techniques, because online forums allow for diverse perspectives on a range of topical issues (Manosevitch and Walker, 2009) within a more naturalistic environment (Jowett, 2015). In relation to alcohol use, one study analysed 758 messages from United Kingdom-based alcohol discussion forums, identifying three themes: sharing, supporting and sobriety (Coulson, 2014). Other studies have analysed reader comments in response to online news articles, e.g., financial incentives for breastfeeding (Giles et al., 2015) and weight loss surgery (Glenn et al., 2012). Growing evidence suggests that online news articles can be key in capturing lay perceptions (Kesten et al., 2014), potentially from those who do not typically participate in research (Holmes et al., 2009).

In 2018, United Kingdom television and radio presenter, Adrian Chiles, made a documentary titled, “Drinkers Like Me”, where he sought medical advice regarding the dangers of his alcohol use. The documentary was also directed at the lobbying power of the alcohol industry and its influence on government policy, yet the government has still not progressed with instituting regulatory measures (Williams et al., 2020). Chiles subsequently wrote an online article for a United Kingdom newspaper, The Guardian^1^, describing his experiences of moderating alcohol consumption since the documentary. There were over a thousand comments in response to the article, from Guardian readers, and this text was used for a secondary qualitative analysis. Using inductive thematic analysis, this study aims to explore (i) posters’ attitudes toward moderating alcohol consumption and (ii) posters’ experiences of moderating or abstaining from alcohol.

## Materials and Methods

### Data Collection

All data were obtained from the comments section of an article titled, “What happened next? ‘Drinking for the sake of drinking. It’s madness’: how Adrian Chiles cut back on booze”, on a United Kingdom news media website, The Guardian (see text footnote 1). Chiles wrote the article as a follow-up to a documentary that he created, titled, “Drinkers Like Me”. In both the documentary and the article, Chiles described his attempts to moderate his alcohol consumption, following medical advice. The documentary and article also explored the impact of not drinking on socialising, alcohol labelling, and problem recognition. The original article was posted at 05.59 GMT on 13th December 2018, with the comment section closing at 20:30 GMT that same day. All comments were obtained for analyses after this date.

The initial number of comments including replies in threads was 1128, of which 364 were original comments (e.g., direct comments on the article) and the remainder were responses to comments. Three coders initially coded all comments, including multiple codes for comments which were irrelevant to the aims of the research. Some examples of irrelevant codes included irrelevant support for Chiles (e.g., “Nice one Adrian. Enjoyed your documentary”), irrelevant abuse/insult (e.g., “Go away Mr Chiles, go cry into your beer”), and random exchange (e.g., “That’s funny!”). As it had been decided *a priori* that irrelevant comments would be removed from the analysis as they would not contribute to the aims of the research, this left *N* = 784 comments and replies.

Comments were copied directly from the website into an excel file in chronological order to assist initial coding. Once comments were coded, these were stored and managed in NVivo 10.0 to assist with further analysis.

### Participants

The Guardian allows the public, including unregistered users, to read articles and comments online. Only registered users may comment on articles, *via* a unique login, and these were pseudo-anonymised to prevent identification.

There were a total of 594 individual posters (i.e., participants) who responded to the selected Guardian article. Due to the nature of this secondary data, it was not possible to determine socio-demographic characteristics of the posters. However, the average characteristics of Guardian readers were aged over 35, of upper to middle social classes with an even gender split (Statista Research Department, 2019).

### Ethics

The University of Liverpool’s ethics committee confirmed that ethical approval was not required for this study and we sought approval from the Guardian for the use of their content and followed the best practice guidelines outlined by the British Psychological Society (BPS). There is limited guidance on the ethics of internet research, as it is relatively novel, therefore it is important to thoroughly address the potential ethical considerations. Most importantly, participants were not able to provide informed consent, as we were unable to identify participants or their personal information to seek consent. However, the BPS states that consent is not needed if the internet communication is considered “public”; the Guardian comments section is publicly available and commenters are made aware that comments can be viewed online and collected by other people (British Psychological Society, 2017; The Guardian, 2018). We were also unable to identify ourselves as researchers, debrief participants, or share findings of the research. Nevertheless, participants were pseudo-anonymised, partial quotations were used, and identifiable information was removed, to prevent any adverse consequences to participants.

### Data Analysis

An inductive thematic analysis was used to identify patterns within the data in relation to the two aims (Braun and Clarke, 2006, 2019). Inductive thematic analysis allows themes and codes to be strongly linked to the data, as opposed to deductive thematic analysis which is primarily theory driven. This involves a six-step process of familiarising with the data, initial coding, generating themes, reviewing themes, defining and naming themes, and writing up the findings.

Upon generating initial codes, researchers then developed a codebook, outlining definitions and examples. After initial coding, codes with their associated extracts were read to identify patterns between the data and codes which resulted in the development of themes and sub-themes. Due to the wealth of data, three female second-year doctoral students (JAP, PI, and JW), who are experienced in conducting qualitative research, each initially analysed a third of all the comments. Data was second coded (10%) to ensure inter-rater reliability. Any discrepancies identified were discussed until all three coders reached a percentage agreement of at least 80%, with Cohen’s kappa above 0.60, indicating at least moderate agreement (McHugh, 2012).

JAP reviewed the codes derived from the data and its associated extracts, to develop themes for the two aims. Memos were also used throughout the analysis among the coders to note salient codes, coders’ thoughts on comments, and potential relationships between the different codes and themes. These memos were also discussed at meetings to inform the development of themes and sub-themes. Main themes were developed by JAP and reviewed, defined, and named by PI and JW and are presented in the results section, using quotations to illustrate each theme.

## Results

For aim one (what are posters’ attitudes toward moderating alcohol?), two themes were developed: “general attitudes toward drinking” and “general attitudes toward reducing consumption”. For aim two (what are posters’ experiences of moderating or abstaining from alcohol?), three themes were developed: “moderation vs. abstinence”, “reflections on past drinking behaviours”, and “current drinking behaviours” (Table 1).

**TABLE 1:** Summary of themes and sub-themes with reference to the relevant research aims.

Theme	Sub-theme	Aim
1. General attitudes toward drinking	1.1. Drinking culture in the United Kingdom vs. other countries 1.2. Perceptions of drinking guidelines and problem drinking	Aim one
2. General attitudes toward reducing consumption		Aim one
3. Moderation vs. abstinence	3.1. Motivations for drinking 3.2. Motivations to reduce consumption 3.3. Positive and negative consequences of reducing consumption	Aim two
4. Reflections on past drinking behaviours		Aim two
5. Current drinking behaviours	5.1. Self-monitoring of drinking behaviours 5.2. Successful strategies to reduce consumption	Aim two

**FIGURE 1:**
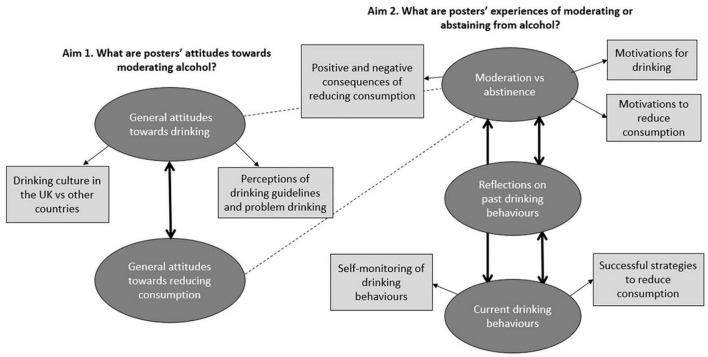
Thematic map outlining the themes (dark grey circles) and sub-themes (light grey rectangles) for each aim. The double-ended arrows represent the linking of themes *within* each aim and the dotted lines represent the linking of themes *across* aims.

The thematic map (Figure 1) presents the themes and sub-themes separately for each aim. The themes *within* each aim are linked because they answer the same respective research question. Some themes *across* the aims are linked, as posters discussed general attitudes toward drinking and reducing consumption, alongside sharing their own experiences Moderation and abstinence were frequently discussed with reference to broader general attitudes and personal experiences, hence the “moderation vs. abstinence” theme linking with both themes for aim one.

### What Are Posters’ Attitudes Toward Moderating Alcohol?

The two themes developed for this aim (“general attitudes toward drinking” and “general attitudes toward reducing consumption”) describe posters’ negative attitudes toward alcohol and the culture of drinking within the United Kingdom, as well as highlighting the perceived societal attitudes toward reducing consumption, through either moderating or abstaining. These themes are linked as they reflect posters’ attitudes toward drinking and reducing consumption, without explicitly sharing their own experiences.

### Theme 1: General Attitudes Toward Drinking

This theme describes the culture of drinking and how it is normalised within society. Overall perceptions of drinking were negative, with many posters discussing the risks of alcohol, which tend to be ignored.

*“By now we’ve all been told how carcinogenic and responsible for so many cancers alcohol is. Why then are SO many people choosing to ignore this message?. unlike smoking, which is widely, unquestioningly and utterly frowned upon.”*

*“Alcohol is addictive and that’s the bit that so many people tend to want to ignore (when it comes to themselves anyhow).”*

This theme consists of two sub-themes: “drinking culture in the United Kingdom vs. other countries” and “perceptions of drinking guidelines and problem drinking”. Posters discussed the current culture of drinking in the United Kingdom, compared to other countries, and how alcohol is normalised, compared to other substances. Misinterpretation of drinking guidelines (0 to 14 units per week) and what constitutes problematic consumption, makes it difficult to address the harms of alcohol and change United Kingdom drinking culture.

#### Sub-Theme 1.1: Drinking Culture in the United Kingdom vs. Other Countries

Posters discussed the drinking culture in the United Kingdom and how it is different to drinking cultures in other countries. Posters described how heavy drinking and binge drinking (to get drunk) are engrained in United Kingdom society and this is perceived as the norm to British people, but in other countries, moderation is more common.

*“The social culture in the* United Kingdom *is very heavily centred on alcohol*…”

*“This country has always had a problem with alcohol. It’s almost treated as a rite of passage to get blotted as soon as possible from a young age, then to carry on as though drinking is an Olympic event for which training is obligatory.”*

*“Fortunately, I moved to Switzerland where if you order a bottle of wine for two – everybody stares – this really helps moderate alcohol intake*… *But it’s true that the drinking culture is very different here than in the* United Kingdom *(where I’m from) – people enjoy a drink or two without the need to get drunk.”*

It was also perceived that in the United Kingdom, but not elsewhere, alcohol is used as a method of coping with stress or low mood.

“…*The problem is not alcohol, it is that the British are miserable and repressed and drink to excess to drown their sorrows*…*.”*

*“The United Kingdom’s problem is not alcohol, it is the shitty stressed lives which the Brits are, quite logically, seeking to escape from.”*

Others note that while heavy drinking is normalised in United Kingdom society, other illicit substance use is disapproved of.

*“The attitude to drinking in Britain is something that needs to be looked at and changed, regular heavy drinking is seen as totally normal, and in a lot of cases it’s actively encouraged. Yet mention other recreational drugs, such as marijuana or ecstasy and there’s uproar and lectures on “gateway drugs.”*

Posters described how United Kingdom drinking culture makes it difficult to change drinking habits, however, making these issues and the benefits of moderation known through the media could help to change this.

*“I’m Spanish but in England for the last 14 years. I am still shocked remembering friends and colleagues telling their weekend stories in the pub: “It was a great weekend I had 11 pints on Saturday”. I can’t see our culture changing*… *but it’s good at least that someone has brought the issue out into the open.”*

#### Sub-Theme 1.2: Perceptions of Drinking Guidelines and Problem Drinking

Posters discussed current drinking guidelines with perceptions of what constitutes problem drinking. The quotes below illustrate that posters are aware of some drinking guidelines yet disregard them.

*“I agree that in general, many people drink too much. I would like to know, however, what is truly a reasonable amount. The 14 units thing I don’t accept, simply because most people lie to the doctor when asked how much they drink, so anything based on that is incorrect.”*

*“Binge drinking is defined as 8 units in a single sitting. Or 4 pints, if you like. That’s really just “drinking”. I always associated “binge” drinking with the likes of George Best or Oliver Reed.”*

While some posters correctly interpreted recommended drinking guidelines, others did not and instead, thought that problem drinking was defined as the frequency and level of alcohol consumption.

*“50 pints a week? Either I’m totally wrong about how much a pint is or that guy knows nothing about alcohol and health. It’s one unit a day, folks. That would be seven pints a week! Let’s face it: if you drink every day, you have a problem. If you drink around 10 units/beer a week you have a problem.”*

Others questioned when drinking becomes a problem, particularly if negative effects were not apparent. Some referred to drinking becoming a habit and losing control, and others referred to interferences with work, as tools for identifying problematic drinking.

*“When is a habit an addiction? Is dependency the same as carrying out a ritual or doing something because you always do? I think Adrian was saying in his documentary that he drank in both social situations and on his own and it was such a habit, that he hadn’t ever questioned whether it was a good or detrimental thing. It was just a repetitive thing, which he clearly enjoyed, but he came to question whether it was having a negative affect on his health, even if he wasn’t dependent as such.”*

This suggests that while some posters are aware of the recommended drinking guidelines, others are not, and instead, assess the frequency or impact of their drinking to determine problematic consumption. This dissonance between drinking guidelines with beliefs around problem drinking may make it difficult for the general population to know when to reduce their consumption. The implications of this may then be compounded when alcohol is embedded within United Kingdom culture, as shown in the previous sub-theme.

### Theme 2: General Attitudes Toward Reducing Consumption

Posters discussed general attitudes toward reducing consumption, through moderation or abstinence. This was discussed in terms of the perceived drinking behaviours of others, where some believed that people are beginning to reduce their consumption, but this may be specific to certain sub-populations, such as the middle class.

“…*Lately, I’m seeing more and more people taking steps to drink less, much less, so really happy seeing Adrian’s column, however, not sure if my sample of society as middle class adopted Londoner is a true reflection of society.*

Overall, posters were supportive of those choosing to reduce their consumption and acknowledged difficulties in doing so, particularly due to the United Kingdom drinking culture in. Original comments by those sharing their own difficulties in moderating generated several replies from others, *via* threads.

*“I’ve a lot of admiration for people who’ve managed to moderate themselves*…”

*“Always amazes me that some people have these strong negative reactions if someone they are with decline a drink or not drink at all.”*

Others highlighted that alcohol is the only activity to apologise for not partaking in, highlighting the need for changes at a societal level.

*“Drinking is addictive, it’s a depressant, it decreases your health, causes cancer, ruins your sleep, skin and teeth, it’s expensive and it makes you fat. It really doesn’t have a lot going for it, and it’s the only drug you have to apologise for not taking.”*

*“The culture of implying that non-drinkers are boring certainly doesn’t help!”*

It was noted that some of the challenges faced when reducing consumption could be addressed by increasing the availability of alcohol-free alternatives or labelling the harms of drinking.

*“What has made no alcohol easier now, is the wider range of tasty non-alcoholic beers. I can look and feel that I’m part of a drinking group and enjoy it.”*

*“…Maybe this is part the problem and that pub culture should become cafe culture along with a restriction on advertising and ‘alcohol kills’ on their labels. Add to that an easing back on those now massive wine and beer displays in supermarkets and a complete covering up of the hard stuff like they do with cigarettes and tobacco.”*

Contrasting this, a few comments suggested that the social environment should not change to accommodate those wishing to moderate or abstain. This illustrates some of the complexities of facilitating a culture change.

*“I’m glad that people who felt that their drinking had become problematic have found a solution but wish that some of them would accept that not everyone who enjoys getting pissed every now and again has a similar problem.”*

*“Open- house on New Year’s Day, a wedding or a birthday party has to be “dry” because an ex-drinker is going to be present? Selfish, or what? Why does the person who can’t do moderation get to dictate things?”*

This theme shows both overarching positive attitudes toward reducing consumption and the challenges of changing a culture of heavy drinking.

### What Are Posters’ Experiences of Moderating or Abstaining From Alcohol?

For this aim, three themes were developed: “moderation vs. abstinence”, “reflection of past drinking behaviours” and “current drinking behaviours”. These themes reflect posters’ own experiences of drinking alcohol (both past and present) and approaches used to moderate their consumption or abstain.

### Theme 3: Moderation vs. Abstinence

This theme describes posters’ experiences of reducing consumption, through either moderating or abstaining. There were differences in preferences for moderating or abstaining, with some believing that moderating is more difficult than abstaining as it requires constant planning and vigilance.

*“I went on the wagon for* + 100 days and felt f*%king A. Then went back to “moderating” and discovered how shit it made me feel. Now it’s abstention. Moderating doesn’t work for me.”

*“Moderating, as opposed to abstaining, is seen as a bit of cop out. Trust me, it isn’t. It requires constant thought; hundreds of decisions have to be made every week.”*

*“I tried moderation but it was just hell on earth, I was much better quitting completely and I’ve never regretted it.”*

This theme consists of three sub-themes, outlining the thought processes when reducing consumption and the implications: “motivations for drinking”, “motivations to reduce consumption” and “positive and negative consequences of reducing consumption.”

#### Sub-Theme 3.1: Motivations for Drinking

Several distinct motivations for drinking were described, with some posters stating that they drink to be sociable or because they enjoy it, whereas others reported drinking to cope.

*“But what else is there? I’m Scottish and single. No drink, no life.”*

*“For me, it’s about drinking because I want to enjoy a single drink*…”

“…*I had a very stressful job in the city, and wine was my relief*…”

Some posters mentioned that drinking had become part of their routine (i.e., habitual) and they were not motivated to change this. This highlights the contrasting experiences within the sample, as not all posters were motivated to reduce their consumption.

*“I still feel I enjoy my drinking, so don’t feel the need to stop.”*

*“A lot of it is habit – and over the last year I’ve changed what I’m used to. Going out for a half is now the norm, and it’s impacted my home consumption too.”*

#### Sub-Theme 3.2: Motivations to Reduce Consumption

Some posters discussed the impact of drinking as a mechanism for change, most notably, damages to health.

*“I realised I needed to moderate after a talking-to from my doctor. I wasn’t obese, or diabetic, or an alcoholic, but if I wasn’t careful, I’d be headed that way, so his warnings were effectively about making life better in future, rather than thinking I could still get away with it all now.”*

Other reasons included maturing out of drinking or worsening hangovers, which may reflect the demographic of Guardian readers.

*“I’m 40 next year and the following day has become such a ghastly ordeal that I’ve pretty much been forced by circumstance to moderate.”*

*“I have been fortunate in that I have gradually lost interest in and taste for alcohol without really thinking about it*…”

Some had relocated abroad which prompted a change, due to differences in drinking cultures, linking with theme 1 (general attitudes toward drinking).

*“I find (through no conscious choice) that I drink far less here. I miss hanging out in my local etc., but that just doesn’t tend to happen here (price is also a major factor), so I don’t find myself in “drinking situations” as often as I did back home in London.”*

#### Sub-Theme 3.3: Positive and Negative Consequences of Reducing Consumption

Posters reported experiencing benefits to changing their behaviours, e.g., weight loss, saving money and leading a more fulfilling life, which encouraged posters to sustain these changes.

*“Just quitting for a matter of weeks has allowed me to see improvements in my health, mood and bank balance. I’ve also taken up jogging and yoga, writing a bit and reading lots. I’m hopeful I can last even longer this time. Good luck to anyone thinking of trying it, well worth it.”*

Some described the negative impact of changing their drinking habits, though this was less common. This links to theme 2 (general attitudes toward reducing consumption) as it highlights the difficulties in reducing consumption when it is not accepted by peers.

*“I was happy enough in myself but had almost no social life and my wife didn’t enjoy drinking alone on holiday.”*

### Theme 4: Reflections on Past Drinking Behaviours

Posters discussed previous drinking habits and acknowledged previously drinking too much, compared with their current drinking habits.

*“I used to drink 50-plus pints a week and loved, wanted and needed each one. But it wasn’t a good idea to carry on like that.”*

*“Been there, done it, still now fighting it and winning to a certain extent*…. *8–10 Stella a night, 6–7 days a week, so that’s easily over 150 units a week.”*

Other posters acknowledged that watching Adrian Chiles’ documentary resonated with their previous drinking habits and compared this to their current drinking habits.

*“This was definitely the most important hour of my year, and possibly my life. I saw this programme and recognised myself*… *Sometimes all you need is a mirror, though I never thought it would be held by Adrian Chiles*…”

Others described the difficulties of knowing when to change their behaviours, especially if there were no obvious consequences.

*“I had an unwritten rule never to drink before 6p.m., functioned at work, now and again a thumping headache, but that wasn’t too often. Ate healthily, cycled, walked, exercised, wasn’t objectionable after drinking, no glaring health-related issues, so it wasn’t a problem, was it? On reflection, it was, and since then I’ve counted the units consumed, and it must have ranged between 30–40 per week.”*

This theme suggests that posters were aware of previous heavy drinking, and this may have informed their current drinking behaviours, hence the link with theme 5 (current drinking behaviours).

### Theme 5: Current Drinking Behaviours

Several posters detailed current drinking behaviours, describing reductions compared to previous drinking behaviours. These were described in relation to the strategies used to reduce their consumption and maintain these behaviours. This theme consists of two sub-themes: “self-monitoring of drinking behaviours” and “successful strategies to reduce consumption.”

#### Sub-Theme 5.1: Self-Monitoring of Drinking Behaviours

Posters reported moderation or abstinence in the context of self-monitoring, relating to themes 3 (moderation vs. abstinence) and 4 (reflection on previous drinking habits). Moderators used different self-monitoring techniques to maintain their reduced consumption, as outlined in the quotes below.

*“I have a very simple rule now – I allow myself no more than a third of a glass of wine or a small beer on week nights.”*

*“I’ve cut down from 7 days a week to 2 evenings a week. This has taken 6 months of slow reduction.”*

Others outlined intentions to self-monitor their drinking behaviours, given this article was published in December 2018, and make changes in the New Year.

*“My plan for next year is to have a fixed number of alcohol free days (thinking of 120 as a target figure) and marking them off as I do them.”*

This sub-theme highlights that some individuals have good awareness of their drinking behaviours, however, there were marked individual differences in perceptions of what constitutes moderating.

#### Sub-Theme 5.2: Successful Strategies to Reduce Consumption

Some posters who changed their drinking behaviours reported changing their drink choice or setting. Some posters described alcohol as an indication of the end of the working day, and therefore, found a substitute helped sustain this.

*“I cut out midweek drinking by having fruit tea, instead. It seemed that was important to me was that a glass of wine signalled that my working day was over*… *Making a cup of tea replaced that line between the difficult part of the day and the easy part and I found that with that, ditching weekday drinking was really easy.”*

Several posters reported drinking alcohol-free beers and there was much discussion surrounding the availability and quality of different alcohol-free beers, *via* threads.

*“There are some really good alcohol-free beers out there, Adnam’s Ghost Ship 0% does an outstanding impression of a real ale, and if you’re into craft beer BrewDog’s “Nanny State” is really quite nice.”*

Others used an alcohol mobile application to track the number of units and to prompt posters when consumption is high.

*“I found the Drink Fee Days app from the NHS to be a great aid. It’s reminders and gentle journey with you help me remember why I am doing this.”*

This theme highlights several successful strategies used to maintain reduced alcohol consumption e.g., self-monitoring units, alcohol-free days, and alcohol-free drinks.

## Discussion

This novel analysis provides an insight into posters’ attitudes toward, and experiences of, moderating or abstaining from alcohol. We identified five key themes and seven subthemes, in response to our two aims. The themes for aim one suggested that posters generally hold negative perceptions of alcohol and were supportive of those choosing to reduce their consumption. Issues surrounding the United Kingdom drinking culture and misperceptions of problematic drinking were discussed. For aim two, the themes represented posters’ own experiences of moderating or abstaining, reflections on previous drinking habits, and the positive and negative consequences of changing such habits. Posters acknowledged the difficulties of reducing consumption, due to normalisation of heavy drinking in the United Kingdom. Misperceptions, or disregard, of current drinking guidelines highlights a need for population-level interventions, to reduce stigma surrounding moderation or abstinence and increase awareness of the dangers of drinking above government guidelines.

Historically, the United Kingdom has been characterised as having a binge-drinking culture (Room et al., 2009). This was reflected in our data, through discussions relating to the normalisation of heavy drinking, particularly in social practices. Shared social norms regarding alcohol can influence consumption at both individual and population levels (McAlaney and McMahon, 2007; Room et al., 2009). Posters generally viewed the United Kingdom drinking culture negatively, suggesting a shift in social norms, particularly for those who may identify with Adrian Chiles, with recent evidence showing a significant increase in the number of males downloading an alcohol reduction app following Chiles’ documentary (Garnett et al., 2021). Celebrities can be influential in raising awareness of health risks as they are viewed as credible sources when communicating information about health consequences (Kaner et al., 2017). Evidence shows that credible sources can be used for effective behaviour change techniques (BCTs; Kaner et al., 2017), such as increasing physical activity (Young et al., 2015). Evidence-based celebrity health promotions (Hoffman et al., 2017) could highlight the dangers of heavy drinking and change social norms which encourage the United Kingdom drinking culture.

Our findings provide support for social practice theory (Nicolini, 2016), which takes the focus away from individual behaviour and focusses more on practices, i.e., performances of routine behaviours which are shared across groups. Within the United Kingdom, drinking practices often occur simultaneously with other practices such as work, eating, or socialising (Meier et al., 2018). Posters shared their own personal struggles with their choice to moderate or abstain from alcohol, particularly in a social context. Although posters were supportive of each other, the lack of support if abstinent or attempting abstinence, from others, was discussed. Interviews with Australian moderators and abstainers showed similar findings, with participants experiencing stigma from others for violating social norms (Bartram et al., 2017a,b). Health promotion initiatives should target the social consequences of reducing consumption.

There were comments which expressed a lack of awareness, or disregard, of current United Kingdom drinking guidelines. This reflects what was observed in a study of United Kingdom drinkers, where 71% were aware of new alcohol guidelines but only 8% knew what they were (Rosenberg et al., 2017). Moreover, United Kingdom drinkers showed no change in their alcohol consumption, following the publication of the revised drinking guidelines (Stevely et al., 2018). A focus group identified that current drinking guidelines were generally disregarded and perceived to be irrelevant, with those who reported moderating alcohol consumption, doing so because of a desire to fulfil work or family responsibilities (Lovatt et al., 2015). Both the original article in the Guardian, and the TV documentary which prompted it, discuss the misperception that drinking above the recommended guidelines is not harmful unless it interferes with personal responsibilities. This indicates a need for a multifactorial intervention approach, at a societal level, to target cultural stigma surrounding moderation or abstinence and increase awareness of the dangers of drinking above government guidelines.

Many posters stated social and enjoyment motivations for drinking, whereas some discussed drinking to cope with a stressful day or drinking because they face pressure from others (Cooper, 1994). Contrarily, posters discussed their motivations to cut down, reporting both internal and external motivations. For some, life events, such as relocation, were external motivations to cut down. This may relate to the maturing out effect (Winick, 1962; Waldorf, 1983; Heyman, 2013). Others reported internal motivations such as physical health or financial consequences of drinking. Little research has explored motivations for reducing consumption, with most focusing on motivations to drink (Cooper, 1994; Kuntsche et al., 2006). Our findings provide a novel insight into reasons why individuals in the United Kingdom may reduce their consumption, in the absence of a diagnosed alcohol problem, which could be used to motivate others to reduce their consumption.

When discussing their own experiences of moderating, posters shared their successful strategies. A range of self-monitoring techniques were mentioned (Kanfer, 1986), such as counting units, or using a smart-phone application to monitor consumption. Evidence suggests that smart-phone applications are useful for reducing alcohol consumption, as they use a range of effective behaviour change techniques, e.g., goal setting and action planning (Garnett et al., 2015). Posters reported changing their drink setting, such as no longer drinking in pubs or at home, which has been shown to be a successful behaviour change technique (Michie et al., 2012; Crane et al., 2015). Alcohol-free drinks were frequently discussed, with threads containing suggestions for alcohol-free beers. Recent United Kingdom statistics showed a 58% increase in alcohol-free beers (Kantar Worldpanel, 2018). Wider availability of alcohol-free drinks may help reduce consumption without altering social practices. The present findings provide insight into successful strategies for reducing consumption which can be used to inform widespread population-based interventions, such as encouraging the use of self-monitoring through smart phone applications.

### Strengths and Limitations

There are several strengths to this secondary analysis of qualitative data. We used a naturalistic method of data collection, as participants were not prompted through questions. The threads of comments can be interpreted as discourse between participants, which occurred naturally without the presence of a researcher, reducing bias (Oswald et al., 2014). Further, we were able to collect a large amount of data in a short time frame, from over 500 pseudo-anonymised posters. However, we only obtained data from one news source and Guardian readers tend to be of high or middle social class and/or over 35, so the findings may be biased by reflecting only the experiences of that demographic. Due to anonymisation concerns, we were unable to examine contextual details such as usernames and avatars which may have provided richer data, in line with a netnographic approach. Moreover, research suggests that individuals who comment on news articles tend to represent a minority of readers, hold more extreme views, and have a particular interest in the topic (Kozinets, 1998, 2015; Purcell et al., 2010; Giles et al., 2015). There is a possibility that the findings were affected by social desirability bias, whereby individuals only commented if they supported the perceptions held in the original article, i.e., believe that moderation or abstinence is important (though examples are provided of posters who wanted to maintain their level of consumption). Further, quasi-quantifiable words (e.g., “some”) were used solely to reflect the commonality of themes and sub-themes within the sample, which cannot necessarily be generalised to the wider population (Lewis et al., 2003; Maxwell, 2010). Nevertheless, the intention of qualitative research is to provide insight and understanding of people’s experiences, and these findings can be used to inform health policies and interventions (Lewin and Glenton, 2018).

## Conclusion

Although alcohol use is heavily embedded within United Kingdom culture, posters were supportive of those who moderate or abstain from alcohol. Posters also acknowledged the difficulties of reducing consumption, particularly from a societal perspective. Adrian Chiles has raised awareness and invoked discussion surrounding alcohol-attributable harms. Future research should investigate the efficacy of celebrity role models in behaviour change interventions, particularly for reducing heavy drinking and encouraging moderation or abstinence within the context of a country’s drinking culture. Our findings highlight the need for public health to focus on shifting alcohol culture, reducing stigma, and increasing availability of alcohol-free alternatives. We present further evidence which can be used to inform government policy and place pressure on the alcohol industry to instate stricter regulatory measures, such as clearer labelling on alcohol products to highlight the associated harms.

## Data Availability Statement

The raw data supporting the conclusions of this article will be made available by the authors, without undue reservation.

## Author Contributions

PI and J-AP contributed equally to the conceptualisation of the study design, data collection, qualitative analysis, the interpretation of the findings, the initial write up of the manuscript, and the revisions. JW contributed to the coding and qualitative analysis. LG developed the initial conceptualisation of the study and the analysis plan. MF, AJ, AKR, SG, and LG provided extensive feedback on the study design, the analyses, the interpretation of the findings, and the manuscript. All authors approved the manuscript as submitted.

## Conflict of Interest

The authors declare that the research was conducted in the absence of any commercial or financial relationships that could be construed as a potential conflict of interest.

## Publisher’s Note

All claims expressed in this article are solely those of the authors and do not necessarily represent those of their affiliated organizations, or those of the publisher, the editors and the reviewers. Any product that may be evaluated in this article, or claim that may be made by its manufacturer, is not guaranteed or endorsed by the publisher.

## Footnotes

1
www.theguardian.co.uk
